# Comparing the vision quality using double-pass technique in eyes with corrected refractive errors and emmetropic eyes

**DOI:** 10.5935/0004-2749.20220050

**Published:** 2025-08-22

**Authors:** Maria A. Henriquez, Samuel-Arba Mosquera, Jorge Camargo, Luis Izquierdo Jr.

**Affiliations:** 1 Research Department, Oftalmosalud Institute of Eyes, Lima, Peru; 2 Biomedical Engineering Office, SCHWIND eye-tech-solutions, Lima Peru

**Keywords:** Refractive error, Emmetropia, Optical device, Diagnostic technique, ophthalmological/instrumentation, Refraction, ocular, Visual acuity, Erro de refração, Emetropia, Dispositivo óptico, Técnica de diagnóstico oftalmológico/instrumentação, Refração ocular, Acuidade visual

## Abstract

**Purpose:**

To evaluate the vision quality by measuring the objective light scatter index and objective optical quality parameters (Strehl Ratio and Modulation Transfer Function) in patients with emmetropia and ametropia.

**Methods:**

This prospective, cross-sectional study included 408 eyes. The ametropic group comprised of eyes with best-corrected visual acuity of 0.0 logMAR or better and present at least a refractive error of ≥0.25 D. Patients underwent slit lamp examination, visual acuity, refraction, and vision quality using the HD Analyzer.

**Results:**

The mean objective light scatter indices were 0.62 ± 0.63, 0.77 ± 0.70, 0.74 ± 0.30, 0.93 ± 0.55, and 0.85 ± 0.61, and mean Strehl Ratio and Modulation Transfer Function scores were 38.17 ± 10.4, 37.37 ± 10.06, 29.84 ± 9.71, 33.2 ± 12.11, and 33.13 ± 10.09 in emmetropes, myopia, hyperopia, spherical equivalent of ≥0, and spherical equivalent of <0, respectively. Differences in all variables were significant between emmetropic and corrected hyperopic and between spherical equivalent of ≥0, and spherical equivalent of <0 eyes (p<0.05).

**Conclusion:**

In spectacle-corrected conditions (with trial frames), emmetropic and simple myopic eyes had significantly better vision quality compared to hyperopic and astigmatic eyes. The clinical significance of these results should be investigated in further studies.

## Abstract (pt)

**Objetivo:**

Avaliar a qualidade óptica medindo o índice de dispersão objetiva de luz e os parâmetros de qualidade óptica objetiva (Razão de Strehl e Função de Transferência de Modulação) em indivíduos com emetropia e ametropia.

**Métodos:**

Estudo prospectivo, transversal, incluindo 408 olhos. O grupo ametrópico era de olhos com melhor acuidade visual corrigida de 0,0 logMAR ou melhor e apresentando, pelo menos, um erro refrativo de 0,25 D ou mais. Os pacientes foram submetidos a exame com lâmpada de fenda, acuidade visual, refração e qualidade óptica com o HD Analyzer.

**Resultados:**

O índice de dispersão objetiva de luz médio foi de 0,62 ± 0,63, 0,77 ± 0,70, 0,74 ± 0,30, 0,93 ± 0,55, 0,85 ± 0,61 e a média da Razão de Strehl e de Função de Transferência de Modulação foram 38,17 ± 10,4, 37,37 ± 10,06, 29,84 ± 9,71, 33,2 ± 12,11 e 33,13 ± 10,09 em olhos emetrópicos, míopes, hipermétropes, equivalente esférico ≥0 e equivalente esférico <0 respectivamente. Foram encontradas diferenças significativas em todas as variáveis entre olhos emetrópicos e com hipermetropia corrigida, equivalente esférico ≥ 0 e equivalente esférico <0 (p<0,05).

**Conclusão:**

Em condições com lentes corrigidas (com armações de prova), os olhos emetrópicos e com miopia simples apresentaram qualidade óptica significativamente melhor em comparação com os olhos hipermétropes e astigmáticos. O significado clínico destes resultados deve ser estudado posteriormente.

## INTRODUCTION

A refractive error indicates a mismatch between the eye’s focal length and its axial length^(1)^, whereas emmetropia would be defined as a perfect match between the eye’s focal length and its axial length. An emmetropic eye usually has excellent uncorrected visual acuity, whereas ametropic eyes usually shows uncorrected visual acuity worse than that of emmetropic eyes due to higher and lower order aberrations, and trial lenses aim to focus the light rays on the retina^(2)^. Being that in both situations, emmetropic and ametropic eyes, the light is focused on the retina, similar visual acuity can be achieved; however, it is uncertain if refractive defect corrected with trial glasses can provide similar vision quality than an emmetropic eye.

Visual acuity is a visual performance measurement based on the spatial resolution of the visual processing system and commonly refers to the vision clarity, but technically rates an examinee’s ability to recognize small details with precision^(3)^. However, vision quality is defined as the unique perception of each individual’s vision; thereby, it is multifactorial, encompassing visual and psychological factors^(4)^. Vision quality is assumed as a better marker than visual acuity for visual performance, since visual acuity is purely a quantitative measure determined under controlled conditions, which does not provide any information on the vision quality nor the total visual capacity in real situations^(5)^.

Recently, the double-pass technique has been proven as a useful tool for measuring high-order aberrations and light scattering^(6)^, a technique that starts from a point light source produced by a laser beam whose image is formed on the retina. When reflected in the retina, the light crosses twice the ocular medium of the HD Analyzer, allowing the evaluation of the retinal image quality using a point projected on the retina, and the size and shape of the reflected light spot are collected and analyzed after the retinal reflex. Images contain all the information about the optical quality of the eye, including higher-order aberrations and diffuse light, which are not usually considered in most aberrometric techniques^(7,8)^. Vision quality has been previously assessed preand postoperatively in cataract, refractive, and corneal surgeries^(9,10)^, showing that refractive surgery allows similar postoperative vision quality when compared with preoperative measurements.

Therefore, this study aimed to evaluate the vision quality of patients with emmetropia and different types of ametropia corrected with trial lenses.

## METHODS

This prospective cross-sectional single-center study included 408 eyes of 408 patients between August and November 2018 at the Oftalmosalud Instituto de Ojos, Lima, Peru. The study complied with the Declaration of Helsinki. The ethics committee of the Oftalmosalud approved the study, and written informed consent was obtained from all participants.

Inclusion criteria for the emmetropic group were as follows: patients who attended the clinic for annual examination; aged between 18 and 45 years; without ocular symptoms or ocular pathology, atopy, irregular corneal patterns, previous ocular surgery, and refractive or refractive error of <0.25 Diopters (D) in the subjective refraction; and with uncorrected distance visual acuity of 0.0 logMAR or better.

The inclusion criteria for the ametropia group were patients who attended the clinic for annual examination; aged between 18 and 45 years; without ocular symptoms or ocular pathology, atopy, irregular corneal patterns, and previous ocular surgery; non-contact lens wearers; with a best-corrected visual acuity (BCVA) of 0.0 logMAR or better; and who presented at least a refractive error of ≥0.25 D on the sphere and/or cylinder. The ametropic group was divided into patients with hyperopic, myopia, and astigmatism according to the subjective refraction. Moreover, astigmatic eyes were subdivided in two groups: spherical equivalent (SEQ) of ≥0 and spherical equivalent of <0.

All patients underwent visual acuity, subjective refraction, slit lamp examination, fundoscopy, and vision quality examinations.

### Vision quality

Vision quality was assessed using the Optical Quality Analysis System (OQAS, HD Analyzer, VISIOMETRICS, Cerdanyola del Vallès, España) by a single trained examiner, only one eye per patient was included in the study that was randomly selected, and the device used an artificial pupil of 4.00 mm in diameter. The head of the patient was positioned on the chin rested and fixated on the center of the figure, and the operator manually aligned the patient’s pupil at the center with the optical axis of the device. In the ametropic group, trial glasses corrected the refractive error, and then the device incorporates a modified Thorner optometer, which is used to compensate for the patient’s residual spherical component, with the optometer range from -8.00 D to +5.00 D. The examiner performed the test and selected the image, only after passing the quality control. Otherwise, the examiner repeated the acquisition until a high-quality image was obtained. With that high-quality acquisition, the device calculates the best refraction for the patient (as a refinement from the trial lenses) and then performs six consecutive measurements based on the previously obtained refraction. These six images are then used to analyze the data and draw a single result for each variable: the Modulation Transfer Function (MTF), the Strehl ratio, and the Objective Scatter Index (OSI)^(11)^. The following variables were measured using the OQAS HD Analyzer:

Objective Scatter Index (OSI) is the ratio between the integrated light in the periphery and in the surrounding areas of the central peak of the double-pass (DP) image. It is based on the analysis of the intensity distribution in the outer parts of the DP image used to quantify the magnitude of intraocular scattering. The OSI is an objective evaluation of intraocular scattered light, and the index is calculated by evaluating the amount of light outside the DP retinal intensity Point Spread Function (PSF) image in relation to the amount of light at the center^(12)^. OSI for normal eyes would range at around 1, whereas values over 5 would represent highly scattered systems^(13)^.

Modulation transfer function cutoff frequency (MTF) is the frequency at which the MTF reaches a value of 0.01, corresponding to a 1% contrast. The value considered is the cutoff point of the MTF curve on the x-axis given in cycles per degree, representing the highest spatial frequency at the lower contrast. The higher the MTF cutoff value, the better the contrast sensitivity^(14)^.

Strehl ratio is an expression of the ratio at the central maximum of the illuminance of the PSF in the aberrated eye to the central maximum found in a corresponding aberration-free system. It is the measure of the fractional drop at the peak of the PSF as a function of the wavefront error. A value of 1 corresponds to a perfect optical system with zero aberration^(15)^.

### Statistical analysis

For each group, data were summarized using the descriptive statistics mean, standard deviation, and range (minimum and maximum value). The comparison of value distribution for each group according to study variables was carried out using the Kruskal-Wallis test and post-hoc Dunn’s test. The linear relationship and correlation among variables were measured using the Spearman’s rank correlation coefficient: a zero coefficient indicates no tendency for Y to either increase or decrease when X increase; for a 3 level system, a value of <0.5 would be considered as weak (<25% variance explained), 0.5 to 0.8 as moderate (25% to 64% variance explained); and >0.8 as a strong correlation (>64% variance explained). All tests were carried out considering a type I error as equal to 0.05, with any p-value of <0.05 being considered statistically significant. All statistical analyses were performed using R *Statistical Software* version 3.4.1 (a free available software under the terms of the Free Software Foundation’s General Public License [https://www.r-project.org/]).

Using the program G * Power version 3.1.9.2 (http://www.gpower.hhu.de/), the power of tests is calculated. All tests reaching a power (1-β) of >0.8 were included for multiple comparisons. For the correlation analysis with a sample size of 23 (the smallest), a power of at least 0.8 was reached for a |r|> 0.7.

## RESULTS

A total of 408 eyes from 408 patients were included: 106 emmetropic eyes and 302 ametropic eyes. Ametropic eyes were divided into three groups according to subjective refraction: 23 simple hyperopia with (+1.38 D ± 0.97 D range 0.25 D to 3.50 D), 32 simple myopia (mean -2.23 D ± 2.08 D, range -0.25 D to -3.50 D), 247 with astigmatism, comprising 91 eyes with an SEQ of ≥0 (0.82 ± 0.79, range 0D to 2.63 D) and 156 eyes with SEQ of <0 (-2.31 D ± 2.33 D, range -10.38 D to -0.13 D).

**Figure 1:**
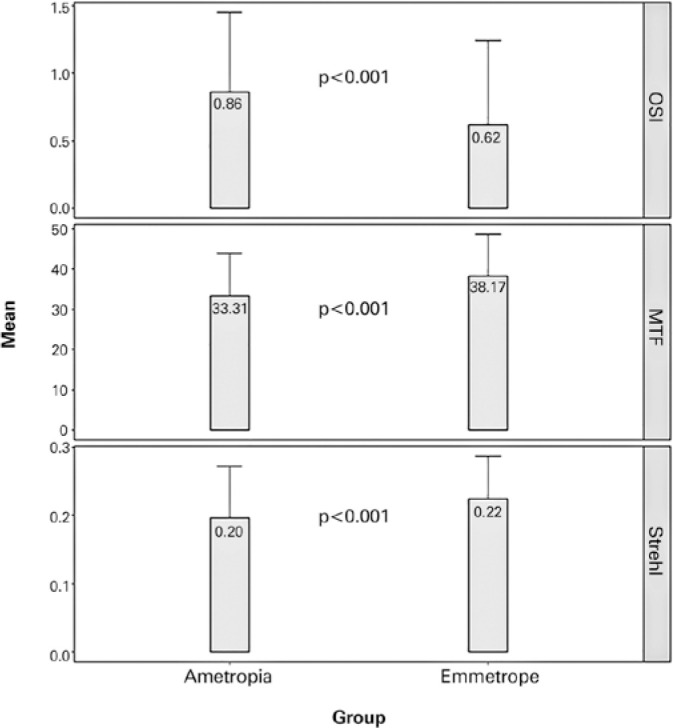
Boxplot showing the quality of vision parameters in the ametropic and emmetropic groups.

Table 1 shows vision quality parameters in emmetropic and ametropic eyes, demonstrating statistically significant differences between them in all the analyzed vision quality parameters. Figure 1 shows that the emmetropic group presented significantly higher Strehl and MTF and lower OSI than the ametropic group (despite the ametropic group was corrected with trial frames achieving BCVA of 0.0 logMAR or better).

**Table 1:** Age and quality of vision parameters in emmetropic and ametropic eyes

Parameters	Emmetrope n=106	Ametropia n=302	P-value^*^
**Age**	31.0 ± 7.8 (18-45)	33.45 ± 8.45 (18-45)	0.13
**OSI**	0.62 ± 0.63 (0.10-3.80)	0.86 ± 0.59 (0.10-3.60)	**<0.001**
**MTF**	38.17 ± 10.40 (13.69-56.99)	33.31 ± 10.67 (11.55-53.65)	**<0.001**
**Strehl ratio**	0.22 ± 0.06 (0.09-0.38)	0.20 ± 0.08 (0.05-0.69)	**<0.001**

OSI= Objective Scattering Index; MTF= Modulation Transfer Function. *P*-value for T- student test between emmetropic and ametropic groups.

OSI= Objective Scattering Index; MTF= Modulation Transfer Function.

**Figure 2:**
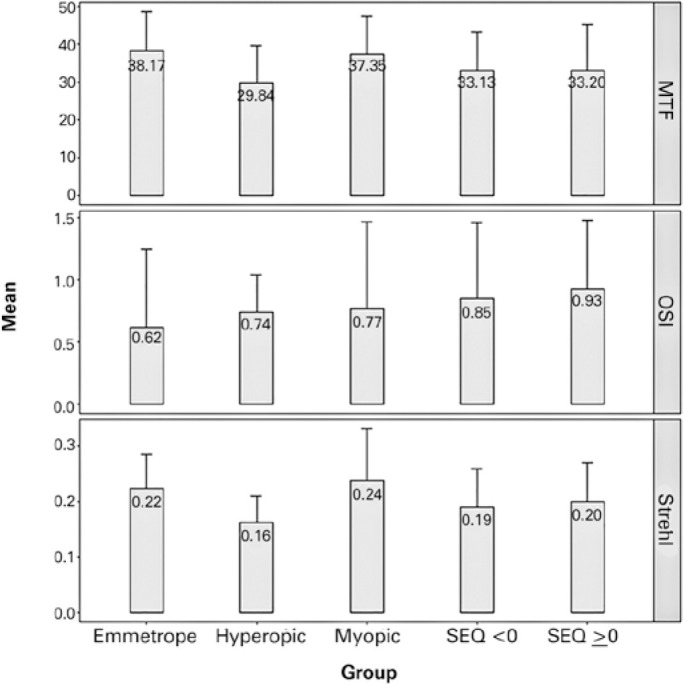
Boxplot showing the quality of vision parameters in the emmetropic, myopic, hyperopic, and astigmatic groups.

**Figure 3:**
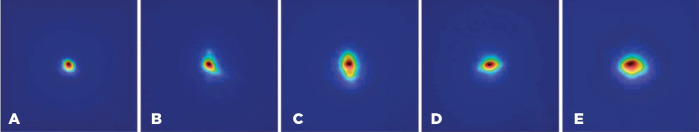
Image displayed by HD Analyzer showing intraocular light dispersion. A. Emmetropia, OSI=0.1; B. Myopia, OSI=0.7; C. Hyperopia, OSI=0.7; D. SEQ ≥0, OSI=1.0; E. SEQ <0 OSI=0.7.

Table 2 shows age and vision quality parameters in different comparison groups. No statistically significant differences were observed between ages in all groups, whereas statistically significant differences were observed in OSI, MTF, and Strehl ratio between the hyperopic and astigmatic groups when compared with emmetropic eyes. No statistical differences were found between the myopic and emmetropic groups. Figure 2 shows that emmetropic group had the higher MTF followed by myopic group but hyperopic group shows lower MTF and also shows that emmetropic group had the lower OSI value and that the myopic group had higher Strehl value followed by emmetropic group but the hyperopic group had the lower Strehl. Figure 3 shows the image displayed by HD Analyzer showing intraocular light dispersion in each subgroup.

**Table 2:** Quality of vision parameters in each studied group

Group	Age	P-value^*^	OSI	P-value^**^	MTF	P-value^***^	Strehl ratio	P-value^****^
**Emmetropes**	31.0 ± 7.8 (18-45)		0.62 ± 0.63 (0.1-3.8)		38.17 ± 10.4 (13.69-56.99)		0.224 ± 0.062 (0.094-0.381)	
**Myopic**	30.9 ± 10.1 (19-45)	0.402	0.77 ± 0.70 (0.2-3.5)	0.2337	37.35 ± 10.06 (19.66-51.78)	0.7189	0.238 ± 0.093 (0.112-0.584)	0.8236
**Hyperopic**	33.7 ± 8.9 (21-45)	0.108	0.74 ± 0.30 (0.3-1.5)	**0.0166**	29.84 ± 9.71 (12.37-47.86)	**0.0035**	0.163 ± 0.047 (0.064-0.268)	**0.0001**
**SEQ** ≥**0**	30.5 ± 8.9 (18-45)	0.159	0.93 ± 0.55 0.10-2.30	**<0.001**	33.2 ± 12.11 11.55-53.65	**0.0036**	0.20 ± 0.07 (0.05-0.49)	**<0.001**
**SEQ <0**	31 ± 8.4 (18-45)	0.179	0.85 ± 0.61 0.20-3.60	**<0.001**	33.13 ± 10.09 12.15-52.70	**0.005**	0.19 ± 0.07 (0.09-0.69)	**<0.001**

OSI= Objective Scattering Index); MTF= Modulation Transfer Function; SEQ= Spherical Equivalent in Astigmatism cases.

*P*-value^*^ between the ametropic group and emmetropic group for age.

*P*-value^**^ between the ametropic group and emmetropic group for OSI.

*P*-value^***^ between the ametropic group and emmetropic group for MTF.

*P*-value^****^ between the ametropic group and emmetropic group for Strehl ratio.

OSI= Objective Scattering Index; MTF= Modulation Transfer Function.

Table 3 shows the Spearman’s rank correlation coefficient [R] value among different ametropic groups (myopia, hyperopia, SEQ ≥0, and SEQ <0) and the vision quality parameters (OSI, MTF, and Strehl). Despite significant correlations were found between magnitude of the ametropia in some groups and some of the vision quality parameters, these correlations were <0.5, representing a weak correlation.

**Table 3:** Spearman’s correlation coefficient between absolute value of the refractive error of all patients and quality of vision parameters

Group	R for OS1	P-value	R for MTF	P-value	R for Strehl	P-value
**Myopic**	0.002	0.991	-0.099	0.590	0.152	0.408
**Hipermetropia**	0.471	**0.023**	-0.284	0.188	-0.294	0.174
**SEQ ≥0**	-0.037	0.730	-0.191	0.073	0.154	0.150
**SEQ <0**	-0.178	**0.025**	0.248	**0.002**	0.181	**0.023**

SEQ= absolute value of the spherical equivalent. (spherical equivalent).

## DISCUSSION

Refractive surgery by any means (corneal or lenticular based) aims for reverting ametropia to emmetropia by adjusting the power of one of the optical elements in the human visual system (cornea or lens) to the axial length of that particular eye. One metric of success can be defined as the difference in visual quality from the preoperative to postoperative status, and an alternative metric can be defined as the difference in visual quality between patients with emmetropes and who underwent post-refractive surgery. However, what is the baseline point of comparison that each person takes as a reference and defines itself as their best visual quality preoperatively, or if this baseline is the same in emmetropes or myopic and hypermetropes corrected with lenses, deduce if postoperative expectations of these patients are related to their baseline, are several questions that this study sought to solve. This work evaluates the difference in visual quality between emmetropes and ametropes using the Optical Quality Analysis System (OQAS, nowadays known as HD Analyzer)^(16)^, and we found significant differences among different groups and subgroups, suggesting that emmetropes and patients with simple myopia achieve higher optical/visual quality than those presenting other ametropias (corrected with trial lenses) when measured with the HD Analyzer. Therefore, emmetropes and patients with simple myopia may be regarded to have “visual optima”.

Similar values and non-significant differences found between emmetropic and myopic groups can be explained in part by previous studies that have reported that the optical quality of the eye derived from wavefront aberration measurements in patients with normal and excellent visual acuity were similar^(17)^. Coma, trefoil, and spherical aberration presented magnitudes up to 0.5 µm, with an average value of approximately zero; eyes with trefoil of >0.25 µm had a high-contrast visual acuity (HCVA) of <1.5. The average optical quality in eyes with HCVA of >1.4 is slightly better than in eyes with normal VA. Moreover, some patients with normal degrees of aberrations attained excellent VA^(17)^. Emmetropic and myopic groups presented lower MTF (higher contrast sensitivity), higher Strehl (lower aberrations), and lower OSI (lower scattering) values than hyperopic and astigmatic groups.

According to our results, emmetropes and patients with simple myopia possess an optical setup that enables not only BCVA of 20/20 or better but also higher optical/visual quality than other patients presenting other ametropias (corrected with trial lenses) when measured with the HD Analyzer. This in turn may indicate that refractive surgery in an ametropic eye (other than simple myopia) may bring the patient’s visual quality closer to that of a naturally emmetropic eye, surpassing the visual quality attained previously with spectacles. Further studies on new populations shall elucidate whether patients who underwent refractive surgery actually present optical qualities (measured by HD Analyzer) better than preoperatively with spectacles (trial lenses) and reach the optical quality of naturally emmetropic patients.

Patients presenting for refractive surgery have an expectation of getting the same postoperative visual acuity and quality without optical aids they had preoperatively with spectacles. The general patient does not have notions of absolute optical quality and performance on the human eye and have their own longitudinal experience. This study shows that the patient with hyperopia and/or astigmatism started “in a disadvantage position” since his/her visual system (spectacles + eye) was less perfect (less optimized) than the that of an emmetropic eye. In other words, this apparent disadvantage may in turn correspond to a clinical opportunity for “underpromizing and overdelivering” (the odds to gain visual quality may be better than for other more optimized conditions such as simple myopia). This may be one of the reasons for decreased satisfaction levels of presbyopic corrections among emmetropic populations^(18)^. Among the groups and subgroups, the simple myopia group showed an optical quality similar to the reference level of the emmetropic population. This may be another reason for the higher level of demand for excellent outcomes in low-to-moderate myopic populations.

Although our study’s quantitative values show a significantly better vision quality in the emmetropic and myopic groups over the hyperopic and astigmatic groups, these results should be cautiously interpreted in the clinical setting. With regard to the measured parameters, OSI of <1 is regarded as normal; therefore, all groups (emetropes, myopia, hyperopia, and astigmatism) in our study actually showed normal OSI values^(19)^, and the obtained MTF cutoff values seem to be on the lower end when compared to the relevant literature^(20)^; however, the mean Strehl ratio was consistent with that in the literature^(15)^.

Some explanations of our findings could be attributed to the fact that different ametropia required different optical profiles in the spectacles (trial lenses) involving different central and peripheral thicknesses. This may be one of the drivers for observed differences, despite the fact that all patients achieved a BCVA of 20/20 or better. For our particular analysis setting, the effect of having corrected patients with spectacles (trial lenses) for assessing the optical quality may have masked and affected some measurements. The HD Analyzer provides an optometer compensator for the defocus term (spherical equivalent of the refraction); however, astigmatism should be corrected with trial lenses. For instance, the visual quality achieved with contact lenses has been known to be better than those achieved with trial lenses^(21)^. Certain types of rigid front-surface aspheric lenses, for example, provide astigmats with even better visual correction than spherical rigid lenses or spectacles; however, the improvement is small and highly patient dependent.

An inadvertent selection bias cannot be excluded in our study among patients who attended to the clinic for annual examination, since they may be patients with “less than normal” visual quality (and thus attend the clinical unit) or may be patients who want to “get rid of their spectacles”. Another limitation of this work is that objective light scatter index, Strehl ratio, and MTF are optical parameters that do not account for the neural process of the visual system^(19)^.

In summary, this study shows that emmetropes and patients with simple myopia achieve higher optical/ visual quality than other patients presenting other ametropias (corrected with trial lenses), and simple myopia corrected with trial lenses had vision quality comparable with emmetropia when measured with the HD Analyzer.
